# Combining rare alleles and grouped pollen donors to assign paternity in pollen dispersal studies

**DOI:** 10.1002/aps3.11330

**Published:** 2020-03-04

**Authors:** Chelsea L. Butcher, Berish Y. Rubin, Sylvia L. Anderson, Vijay K. Nandula, Micheal D. K. Owen, Randolph G. Gardner, J. D. Lewis

**Affiliations:** ^1^ Louis Calder Center, Biological Field Station Fordham University 31 Whippoorwill Road Armonk New York 10504 USA; ^2^ Center for Urban Ecology Fordham University 441 East Fordham Road Bronx New York 10458 USA; ^3^ Department of Biological Sciences Fordham University 441 East Fordham Road Bronx New York 10458 USA; ^4^ Department of Mathematics and Natural Sciences Northwood University 4000 Whiting Drive Midland Michigan 48640 USA; ^5^ Crop Production Systems Research Unit, Agricultural Research Service U.S. Department of Agriculture 141 Experiment Station Road Stoneville Mississippi 38776 USA; ^6^ Department of Agronomy Iowa State University 716 Farm House Lane Ames Iowa 50011 USA; ^7^ Department of Horticultural Science North Carolina State University Mountain Horticultural Crops Research and Extension Center (MHCREC) 455 Research Drive Mills River North Carolina 28759 USA

**Keywords:** Amaranthaceae, experimental arrays, gene flow, paternity assignment, pollen dispersal, Solanaceae

## Abstract

**Premise:**

Pollen dispersal plays a critical role in gene flow of seed plants. Most often, pollen dispersal is measured using paternity assignment. However, this approach can be time‐consuming because it typically entails genotyping all pollen donors, receptors, and offspring at several molecular markers.

**Methods:**

We developed a faster, simpler protocol to track paternity, using pollen receptors and grouped pollen donors that possess rare alleles. We tested this approach using wind‐pollinated *Amaranthus tuberculatus* and insect‐pollinated *Solanum lycopersicum*. After screening potential markers for rare alleles, we grew both species in experimental arrays under field conditions.

**Results:**

All tested *A*. *tuberculatus* seeds and 97% of *S*. *lycopersicum* fruits could be assigned to the grouped pollen donors using each of two markers. From these results, we could infer paternity of untested offspring and assess pollen dispersal patterns in each array.

**Discussion:**

By combining rare alleles and grouped pollen donors, we could assess pollen dispersal for both species and across all arrays after genotyping a small number of pollen donors and a representative subset of offspring. While directly applicable to *A. tuberculatus* and *S. lycopersicum*, this approach could be used in other species to assess pollen dispersal under field conditions.

Pollen dispersal plays a key role in population dynamics and gene flow in seed plants because of its role in fertilization (Ennos, 1994). Consequently, several methods have been developed to measure pollen dispersal (Smouse et al., 2001; Ashley, 2010; Van Rossum et al., 2011). These methods typically involve either trapping or tracking pollen grains. For example, pollen traps (Millwood et al., 2017), fluorescent‐dyed pollen analogues (Waser and Price, 1982; Van Geert et al., 2010, 2014; Van Rossum et al., 2011), and tracking pollinator movements (Pasquet et al., 2008) are widely used to assess pollen movement. However, each approach has logistical or methodological constraints that limit their utility for assessing pollen movement patterns (Thomson et al., 1986; Adler and Irwin, 2006; Pasquet et al., 2008; Ashley, 2010). Critically, they do not allow for measurement of gene flow, because effective pollen dispersal (i.e., dispersal that results in successful fertilization) is not captured (Streiff et al., 1999).

Effective pollen dispersal has been inferred in studies via the two‐generation analysis model (i.e., TwoGener; Austerlitz and Smouse, 2001; Smouse et al., 2001) or neighborhood mating model (Adams and Birkes, 1991; Burczyk et al., 2006; Chybicki and Burczyk, 2010). TwoGener uses genotypic data from pollen receptor plants (i.e., maternal plants) and offspring to estimate the mean effective pollen dispersal distance and number of effective pollen donors (Smouse et al., 2001; Hardy et al., 2004; Sork and Smouse, 2006). Neighborhood mating models also require genotyping the pollen receptor and offspring, as well as potential pollen donors (i.e., paternal plants; Chybicki and Burczyk, 2010). These data, too, are used to calculate pollen dispersal parameters, including the rate of dispersal from pollen donors outside the study area (Chybicki and Burczyk, 2010). However, neither method can be used to directly measure effective pollen dispersal parameters for individual pollen (Smouse and Sork, 2004).

Paternity assignment, or assigning each offspring to a pollen donor, is the only method for directly measuring effective pollen dispersal (Ashley, 2010). As with neighborhood mating models, paternity assignment requires genotyping pollen receptor plants, offspring, and potential pollen donors (Sork et al., 1999; Streiff et al., 1999). More molecular markers may be needed with this method, however, as paternity assignment inherently requires assigning paternity for all offspring (Burczyk et al., 2006). The number of markers required varies (Ashley, 2010), but as many as 18 markers have been used (Buehler et al., 2012; Moracho et al., 2016). Microsatellite DNA markers are used most often, as these loci typically are highly polymorphic and codominant, thus allowing paternity assignment to a specific pollen donor (Wu et al., 1994; Selkoe and Toonen, 2006). The need to develop and optimize primers for a large number of DNA markers, coupled with genotyping all offspring and potential pollen donors, and then using an assignment program to assign paternity, makes this method far less efficient than other methods (Austerlitz and Smouse, 2001; Van Rossum et al., 2011).

Here, we demonstrate a more efficient approach to measuring pollen dispersal in an experimental array by using paternity assignment that combines two methods typically used separately: physically grouping experimental pollen donors near the center of the array (e.g., Lavigne et al., 1998; Jhala et al., 2011; Sarangi et al., 2017) and identifying paternity using rare alleles (e.g., Müller, 1977; Ennos and Clegg, 1982; Krauss, 1994). By using experimental pollen donors and receptors that possess rare alleles likely to be absent in local populations (i.e., populations outside of the experimental array), we could assign paternity without the need to genotype potential donors from local populations and, by grouping experimental pollen donors, we could attribute pollen dispersal and gene flow to pollen donors from a specific location. We tested this approach by identifying rare alleles at a small suite of molecular markers in two populations of the wind‐pollinated common waterhemp (*Amaranthus tuberculatus* (Moq.) J. D. Sauer) and one breeding line of the insect‐pollinated tomato (*Solanum lycopersicum* L.). We then assessed the effectiveness of these markers for measuring pollen dispersal by assessing paternity of seeds produced in naturally pollinated experimental arrays of *A. tuberculatus* and *S. lycopersicum* pollen donor and receptor plants. For each species, we further tested whether rare alleles from different genes differed in their effectiveness in assigning paternity.

## METHODS

### Study species

*Amaranthus tuberculatus*, which includes common and tall waterhemps but is described as a single species (Pratt and Clark, 2001), was chosen as the wind‐pollinated species because members of this genus grow readily in urban environments (Aloisio et al., 2016). Furthermore, this species is dioecious (Hager et al., 1997), which allowed us to assess pollen dispersal to female from male plants, with female and male plants serving as pollen receptors and donors, respectively. To use pollen receptors and donors likely to have rare alleles that are not found locally (i.e., populations outside of the experimental array), we obtained seeds from an Ames, Iowa (USA), population (Hinz and Owen, 1997) and from a Washington County, Mississippi (USA), population (Nandula et al., 2013). These populations were both >1500 km from our study sites.

*Solanum lycopersicum* was chosen as the insect‐pollinated species because, as with *A. tuberculatus*, members of the genus grow in urban areas (Del Tredici, 2010). Although this species typically self‐fertilizes (Rick, 1988, 1995; Chen and Tanksley, 2004), the NC4 grape breeding line is heterozygous for the *ms‐10* male‐sterility gene (Panthee and Gardner, 2013), such that it produces both male‐sterile and male‐fertile offspring. Because male‐sterile flowers do not produce pollen, we could assess pollen dispersal by using male‐sterile plants as receptors and male‐fertile plants as donors. The NC4 line also possesses resistant alleles at two genes associated with resistance to two pathogens: (1) tomato spotted wilt virus (*Sw‐5* gene) and (2) Fusarium wilt race 3 (*I‐3* gene). It is unlikely that *S. lycopersicum* varieties sold in our study area possess resistant alleles at both genes, because the associated diseases are not problematic in the New York metropolitan area. Therefore, local plants should only possess the susceptible alleles.

### Greenhouse methods

Seeds were stored at 4°C until germination. *Amaranthus tuberculatus* and *S. lycopersicum* were grown in 2014 and 2015, respectively, following the same protocols except as noted. To break dormancy, the Iowa *A. tuberculatus* seeds were cold‐stratified in damp filter paper at 4°C for 14 d, then dried at 25°C for 48 h immediately prior to germination. No other seeds were cold‐stratified. Seeds were germinated in Sun Gro Sunshine soil mix #4 (Sun Gro Horticulture, Agawam, Massachusetts, USA) with Osmocote 14‐14‐14 fertilizer (Scotts Miracle‐Gro, Marysville, Ohio, USA) in 53 × 28 × 5‐cm (l × w × d) potting trays (Griffin Greenhouse Supplies, Tewksbury, Massachusetts, USA) in a greenhouse at the Louis Calder Center, Fordham University's field station in Armonk, New York, USA. Every other day, trays were bottom‐watered with well water until the soil was saturated. When seedlings collectively reached 5–10 cm tall (measured from soil to apical meristem), they were transplanted to 10‐cm‐diameter (0.57‐L) pots (Griffin Greenhouse Supplies) with Sun Gro Sunshine soil mix #4 and watered every other day with well water to through‐flow. Six weeks after germination, tissue was collected from 1–2 young leaves on each plant, and a randomly selected subset of samples (12 *A. tuberculatus*, 18 *S. lycopersicum*) was used to identify paternity assignment markers.

Prior to placing the plants in the experimental arrays, plants were confirmed as pollen donors or receptors by flower morphology, inflorescences were removed to minimize seeds or fruits produced as a result of pollination while in the greenhouse, and plants were transplanted to 11.4‐L pots (Griffin Greenhouse Supplies) with Sun Gro Sunshine soil mix #4; these plants served as the parent plants in the experimental arrays.

### Marker development

For *A. tuberculatus*, we assessed seven potential markers (five from *A. tuberculatus* and one each from *Atriplex covillei* J. F. Macbr. and *Suaeda vermiculata* Forssk. ex J. F. Gmel. [also in the Amaranthaceae]): internal transcribed spacer (ITS) region, putative DEAD box ATP‐dependent RNA helicase (*PutDead*) gene, putative glutaredoxin‐like protein (*PutGlu*) gene, putative subtilisin‐like protease‐like protein (*PutSub*) gene, putative transmembrane 9 superfamily 4‐like protein (*PutTrans*) gene, external transcribed spacer (ETS) region, and phosphoenolpyruvate carboxylase (*PEPC*) gene (Table 1). We identified all sequences using the GenBank PopSet database (https://www.ncbi.nlm.nih.gov/popset) and manually designed forward and reverse primers for each sequence. Primers were synthesized with T7 added to the 5′ end of the forward primers and Sp6 added to the 5′ end of the reverse primers (Invitrogen, Thermo Fisher Scientific, Waltham, Massachusetts, USA). Although our goal was to optimize primer pairs for a single marker, we created primer pairs for seven markers because we assumed that (1) we would not be able to optimize all primer pairs; (2) some markers would be monomorphic or share genotypes between populations, such that we would not be able to assign paternity; and (3) some markers would be too polymorphic within a population to allow for easy paternity assignment.

**Table 1:** Molecular markers screened as possible *Amaranthus tuberculatus* paternity assignment markers, GenBank accessions, primer sequences, and expected product size.^a^

Marker	GenBank accession no.	Primer sequences (5′–3′)	Expected product size (bp)
ITS	KC747449.1 in PopSet 583871628	F: TGCCTAGCAGATTGACCAG	517
		R: GATTGCATTCTAGGCTAGG	
*PutDead*	KC747280.1 in PopSet 583871193	F: GATCGTGAGTCTACCTTAG	684
		R: GGTAACTTTCCAACGAG	
*PutGlu*	KC747394.1 in PopSet 583871485	F: AGCTCTTCGTTGACTTCC	467
		R: CCTTGTCTGACTTCTTCG	
*PutSub*	KC747337.1 in PopSet 583871337	F: TCAGGGACAAAGCTTGG	678
		R: CAACCTTCACTGATGTG	
*PutTrans*	KC747224.1 in PopSet 583871044	F: GATCGGAGATGGTGTCC	667
		R: GTAGACAGCAACAGAACC	
ETS	HM005799.1 in PopSet 311235726	F: AGGATCAACCAGGTAGC	383
		R: GCTCCATGCTTGTGCATC	
*PEPC*	KF964102.1 in PopSet 597501860	F: TCGAGAGTGGTCAGAGGAG	774
		R: CATAGGAGAACTTACACGACG	

Note

ETS = external transcribed spacer region; ITS = internal transcribed spacer region; *PEPC* = phosphoenolpyruvate carboxylase gene; *PutDead* = putative DEAD box ATP‐dependent RNA helicase gene; *PutGlu* = putative glutaredoxin‐like protein gene; *PutSub* = putative subtilisin‐like protease‐like protein gene; *PutTrans* = putative transmembrane 9 superfamily 4‐like protein gene.

^a^ For the ITS, *PutDead*, *PutGlu*, *PutSub*, and *PutTrans* markers, primers were created from *A. tuberculatus* sequences. For the ETS marker, primers were created from an *Atriplex covillei* sequence, and for the *PEPC* marker, primers were created from a *Suaeda vermiculata* sequence. Both *A. covillei* and *S. vermiculata* are members of the Amaranthaceae family.

For *S. lycopersicum*, we evaluated two primer pairs for each disease resistance gene (i.e., the *Sw‐5* gene for tomato spotted wilt and the *I‐3* gene for Fusarium wilt race 3). These included one published primer pair (Dianese et al., 2010) and one primer pair from Samuel Hutton at the University of Florida (personal communication) for *Sw‐5* markers, and two published primer pairs (Li et al., 2018) for *I‐3* markers (Table 2). Although our goal was to optimize one primer pair per marker for the NC4 line, we initially identified two primer pairs per gene because, although these primer pairs had been optimized for several *S. lycopersicum* breeding lines (Dianese et al., 2010; Li et al., 2018; Samuel Hutton, personal communication), they had not been optimized for the NC4 line. Primers were synthesized with T7 added to the 5′ end of the forward primers and Sp6 added to the 5′ end of the reverse primers (Invitrogen, Thermo Fisher Scientific).

**Table 2:** Molecular markers screened as possible *Solanum lycopersicum* paternity assignment markers.

Marker (primer pair name)	Citation^a^	Primer sequences (5′–3′)	Expected product size (bp)
*Sw‐5* (*UF_Sw‐5)*	Samuel Hutton, University of Florida, pers. comm.	F: GCGTCATGAAGTCCCACTTT	R = 132, S = 108
		R: GGACTGGTGATGTGCAGGTA	
*Sw‐5* (*Sw‐5_2*)	Dianese et al., 2010	F: AATTAGGTTCTTGAAGCCCATCT	R = 530, S = 420 or 466
		R: TTCCGCATCAGCCAATAGTGT	
*I‐3 (I‐3_7g501*)	Li et al., 2018	F: ACTCTGTCCACCAAAGCTCAA	R = 106, S = 89
		R: TGATTTTTCAATTTTCAGGCTTC	
*I‐3* (*I‐3_7g595*)	Li et al., 2018	F: CATTTAGTCAGACGGCTAATGA	R = 118, S = 104
		R: TTGAGTTTGGTGTTTAAATTGGA	

Note

R = resistant allele; S = susceptible allele.

a Citations where primers were first identified.

To optimize these primers, we extracted DNA from one leaf from each of six *A. tuberculatus* plants from the Iowa and Mississippi populations (three female and three male from each population). For *S. lycopersicum*, we extracted DNA from one leaf from each of nine male‐sterile and nine male‐fertile plants, as well as from a cultivar at the New York Botanical Garden (Bronx, New York, USA). Because this sample was collected locally, it was expected to be homozygous for the susceptible allele at both markers. Before all DNA extractions, leaf tissue was disrupted in Lysing Matrix D using a Fast Prep‐24 instrument (MP Biomedicals, Santa Ana, California, USA) at 6 m s^−1^ for 40 s. Then, DNA was extracted using a DNeasy Plant Mini Kit (QIAGEN, Hilden, Germany), following the manufacturer's protocol modified with a longer (30 min) initial incubation step.

For both species, each PCR cocktail contained 12.5 μL of GoTaq Green Master Mix (Promega Corporation, Madison, Wisconsin, USA), 0.2 μM of forward primer (0.5 μL), 0.2 μM of reverse primer (0.5 μL), 1 ng of template DNA (1 μL), and 10.5 μL of dH_2_O. Each PCR began with a denaturation step at 94°C for 4 min, then 35 cycles of 30 s at 94°C, 30 s at 58°C, and 30 s at 72°C. The samples were then held at 72°C for 4 min to allow for a final extension, and finally at 4°C. Primer pairs for six *A. tuberculatus* markers (ITS, *PutDead*, *PutGlu*, *PutSub*, *PutTrans*, and ETS) were optimized at these conditions.

For the other five primer pairs, we tested a range of PCR conditions by adjusting annealing temperatures (±4°C), the number of cycles (up to 42 cycles), and DNA concentration (up to 5 ng). The *UF_Sw‐5* primer pair was optimized with an annealing temperature of 57°C, 1 ng of DNA, and 40 PCR cycles. The *I‐3_7g501* primer pair was optimized with an annealing temperature of 55°C, 1 ng of DNA, and 40 PCR cycles. We were unable to optimize the primer pair for the *PEPC* gene marker, a primer pair for an *Sw‐5* marker (*Sw‐5_2* primer pair), and a primer pair for an *I‐3* marker (*I‐3_7g595* primer pair) within these parameters. However, as we were able to optimize another primer pair for the *Sw‐5* marker (*UF_Sw‐5*) and another primer pair for the *I‐3* marker (*I‐3_7g501*), these markers were still represented in the paternity assignment. The *PEPC* marker was dropped from further analysis.

All *A. tuberculatus* PCR products were run on 1% agarose gels to confirm the presence of PCR product, whereas the *S. lycopersicum* PCR products were visualized on 3% agarose gels. These PCR products were run on denser gels because the resistant and susceptible *Sw‐*5 and *I‐3* gene marker alleles differ in size, allowing differentiation of the two alleles on 3% agarose gels (Samuel Hutton, personal communication). For verification, we also sent these PCR products to GENEWIZ (South Plainfield, New Jersey, USA) or Macrogen (Rockville, Maryland, USA) for PCR product purification and Sanger sequencing. Sequences were manually edited and aligned in Geneious version 7 (Biomatters, Auckland, New Zealand).

### Experimental arrays

To study pollen dispersal under field conditions, we grew *A*. *tuberculatus* in two arrays of receptor and donor plants in 2014, and *S*. *lycopersicum* in four arrays of receptor and donor plants in 2015. The *A*. *tuberculatus* arrays were placed on a lawn at the Louis Calder Center (Calder) in Armonk, New York, USA, and the roof of the four‐story parking garage at Fordham University's Rose Hill Campus (Rose Hill) in Bronx, New York, USA. *Solanum lycopersicum* arrays were placed at Calder and Rose Hill, plus the green roof of the four‐story Javits Convention Center (Javits) in New York, New York, USA, and a lawn at the Queens Zoo (Queens Zoo) in Queens, New York, USA. At each site, we surveyed an approximate 150‐m‐radius circle around each array to determine if either species was present. While *S. lycopersicum* plants were present in the area around two of the four *S. lycopersicum* experimental arrays (i.e., Rose Hill, Queens Zoo), we did not detect *A. tuberculatus* in the area surrounding the two *A. tuberculatus* arrays.

**Figure 1:**
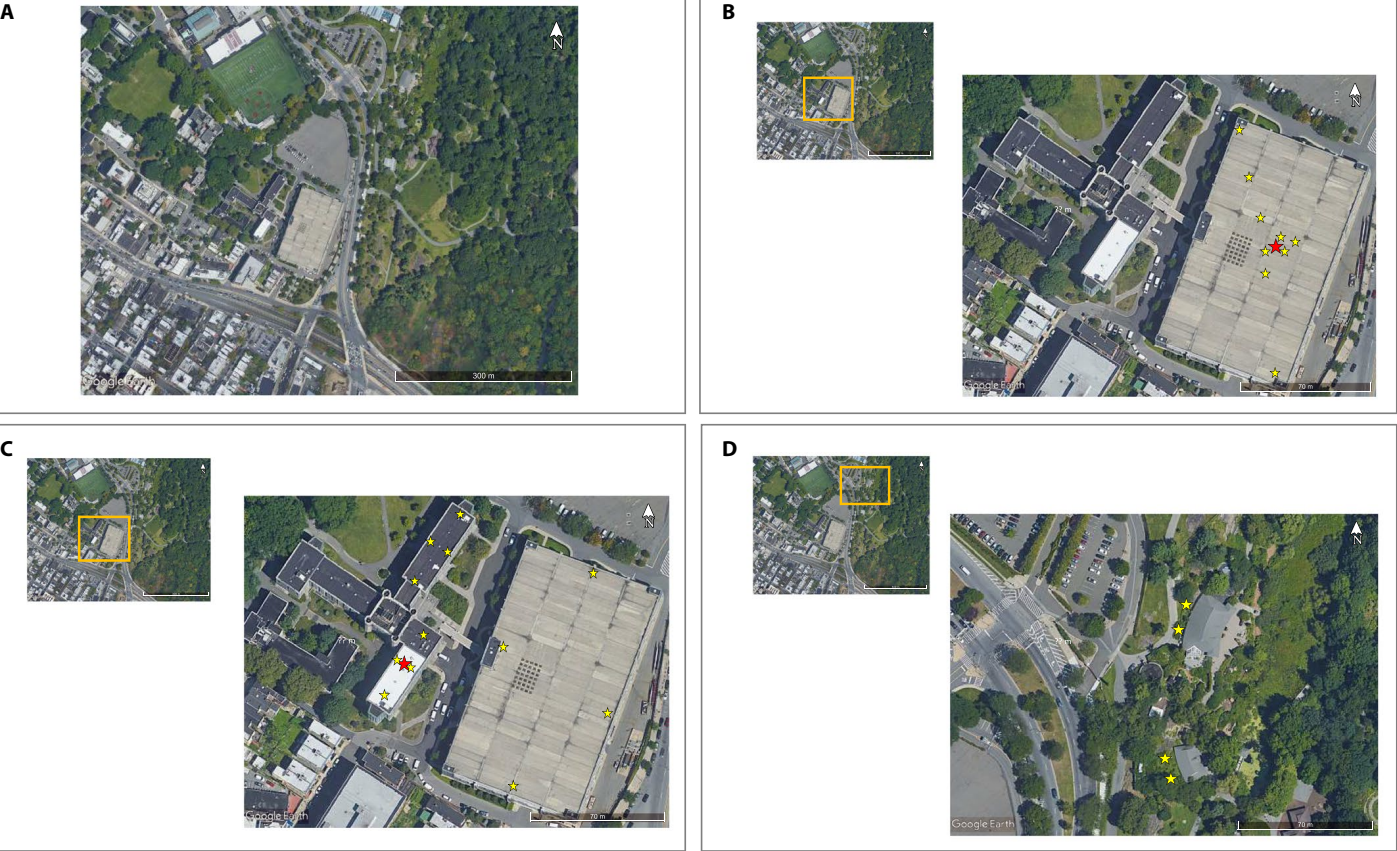
Satellite images of the Rose Hill site, showing an overview of the entire Rose Hill site (A), the experimental array layout for *Amaranthus tuberculatus* (B), and the experimental array layout for *Solanum lycopersicum* (C, D). In B–D, the red star denotes the pollen donor group and the yellow stars each denote a pair of pollen receptors.

At each site, 12 *A. tuberculatus* male plants (in 2014) and 12 male‐fertile *S. lycopersicum* plants (in 2015) were physically grouped in two rows of six plants at the center of each array (i.e., pollen donor group). Pollen receptor plants were arrayed in pairs at increasing distances from the pollen donor group, starting at 1 m from the donor group. Although the array configuration was similar between species and among sites, the number of receptor plants and the maximum distance between the donor and receptor plants depended on the species, as well as the shape of each site (Table 3). To juxtapose the arrays for both species at one site, the Rose Hill arrays are shown in Fig. 1. Array configuration was chosen assuming isotropic pollen dispersal; thus, the design did not include receptor plants at matching distances in each direction. The arrays were maintained for eight weeks. Plants were watered with untreated tap water three times per week to through‐flow and allowed to pollinate naturally. During week 2, plants were watered to through‐flow with 5 cm^3^ per 3.8‐L tap water mixture of Miracle Grow Bloom Booster 10N‐52P‐10K (Scotts Miracle‐Gro) to promote flowering.

**Table 3:** Number of pollen receptor plants, and the maximum distance between pollen receptor plants and the pollen donor group by site and species.

Site/Species	No. of PR plants	Maximum distance between PD group and PR plants (m)
Rose Hill		
*Amaranthus tuberculatus*	18	64
*Solanum lycopersicum*	32	340
Javits		
*Solanum lycopersicum*	16	200
Calder		
*Amaranthus tuberculatus*	16	32
*Solanum lycopersicum*	24	190
Queens Zoo		
*Solanum lycopersicum*	22	146

Note

PD = pollen donor; PR = pollen receptor.

At the end of the study period, *A. tuberculatus* seeds were collected en masse and stored in 5.72 × 8.89‐cm coin envelopes until germination. *Solanum lycopersicum* fruits were collected as they ripened, to minimize the effects of frugivory on estimates of pollination. Seeds were then removed from the fruits and placed on 125‐mm‐diameter, grade 1 Whatman cards (GE Healthcare Life Sciences, Pittsburgh, Pennsylvania, USA). After the pericarp surrounding the seeds had dried at room temperature (about 24°C), thus fastening the seeds to the cards, they were stored at room temperature until germination. Any parthenocarpic fruits were excluded from the analyses because these fruits were not produced as the result of a fertilization event.

### Paternity assignment

All molecular techniques used for paternity assignment followed the protocols described under “Marker Development,” except that DNA extractions from hypocotyl and cotyledon tissue (*A. tuberculatus*) required a 2‐h initial incubation step.

We genotyped all *A. tuberculatus* pollen receptor and donor plants (leaf tissue) and hypocotyl and cotyledon tissue from up to 20 germinated seeds per receptor using the ETS and *PutDead* markers. Specifically, seeds were germinated on damp filter paper at 37°C, and DNA was extracted from hypocotyl and cotyledon tissue (~4 cm). We then used simple exclusion (similar to Ellstrand, 1984) to confirm that pollen dispersed from an experimental donor and not a local donor. In other words, paternity assignment involved subtracting the known maternal genotype from the seed genotype, then comparing the seed's paternal genotype to the experimental pollen donor groups’ genotype(s).

For *S. lycopersicum* paternity assignment, we genotyped all pollen receptor and donor plants, as well as two randomly chosen seeds from each fruit produced at the two farthest distances and two randomly chosen seeds from two randomly chosen fruits produced at two additional randomly chosen distances at both the *Sw‐5* and *I‐3* markers. Specifically, these seeds were germinated in the greenhouse in 10‐cm‐diameter (0.57‐L) pots with Sun Gro Sunshine soil mix #4 and bottom‐watered twice per week with well water until the appearance of the first true leaf, which was collected for DNA extraction. Because all *S. lycopersicum* plants in the arrays carried the resistant alleles, while local plants should only carry the susceptible alleles, seeds fertilized by a local pollen donor could be detected by the presence of both the resistant and susceptible alleles in the PCR product. We predicted that, if we did not find evidence of fertilization from a local pollen donor in seeds produced at the two farthest distances and two randomly chosen distances, we were unlikely to detect fertilization from local donors at distances closer to the experimental donor group. If we found evidence of fertilization from a local donor at a given site, we also assigned paternity to two randomly chosen seeds from two randomly chosen fruits from every receptor plant at that site to survey the degree of pollen dispersal from local pollen donors. We genotyped more than one seed per fruit because, although multiple paternity within a single fruit has not been investigated in *Solanum*, it has been observed in other species (e.g., *Ipomopsis aggregata* [Campbell, 1998], *Silene latifolia* [Teixeira and Bernasconi, 2007]).

### *Amaranthus tuberculatus* markers

The ETS marker (385 bp) exhibited only one genotype in each population, with five single‐nucleotide polymorphisms (SNPs) between the Iowa and Mississippi populations, three of which were homozygous, making this marker ideal for paternity assignment (Table 4, Appendix S1). Because the ETS and ITS regions are both within introns of the 18S‐5.8S‐25S rDNA genes, we excluded the ITS marker (517 bp) from further consideration, so that other usable markers would be from different loci. The other four markers for which we were able to optimize PCR conditions (*PutSub*: 678 bp, *PutGlu*: 467 bp, *PutTrans*: 668 bp, and *PutDead*: 684 bp) were all polymorphic. For *PutGlu*, the two populations shared one genotype, which made differentiating populations challenging. For *PutSub*, the two populations did not share any genotypes, but the presence of numerous polymorphisms made paternity assignment difficult. Therefore, the *PutGlu* and *PutSub* markers were not utilized.

**Table 4:** GenBank accession numbers by genotype for the three *Amaranthus tuberculatus* markers and the two *Solanum lycopersicum* markers identified with rare alleles usable for paternity assignment for the respective species.

Species	Marker	Genotype	GenBank accession no.
*Amaranthus tuberculatus*	ETS	Iowa population genotype	MN698701
		Mississippi population genotype	MN698702
*Amaranthus tuberculatus*	*PutTrans*	Iowa population genotype 1	MN698686
		Iowa population genotype 2	MN698687
		Iowa population genotype 3	MN698688
		Mississippi population genotype 1	MN698689
		Mississippi population genotype 2	MN698690
		Mississippi population genotype 3	MN698691
		Mississippi population genotype 4	MN698692
*Amaranthus tuberculatus*	*PutDead*	Iowa population genotype 1	MN698693
		Iowa population genotype 2	MN698694
		Iowa population genotype 3	MN698695
		Iowa population genotype 4	MN698696
		Mississippi population genotype 1	MN698697
		Mississippi population genotype 2	MN698698
		Mississippi population genotype 3	MN698699
		Mississippi population genotype 4	MN698700
*Solanum lycopersicum*	*Sw‐5*	Resistant	MN698682
		Susceptible	MN698683
*Solanum lycopersicum*	*I‐3*	Resistant	MN698684
		Susceptible	MN698685

Note

ETS = external transcribed spacer region; *PutDead* = putative DEAD box ATP‐dependent RNA helicase gene; *PutTrans* = putative transmembrane 9 superfamily 4‐like protein gene.

The two remaining markers produced results suggesting they could be used for paternity assignment. For *PutTrans*, the Iowa population exhibited three genotypes, none of which were identical to any of the four genotypes within the Mississippi population (Table 4, Appendix S2). For *PutDead*, each population exhibited four genotypes, but the two populations did not share any genotypes (Table 4, Appendix S3). Furthermore, both markers had a similar number of SNPs between each population (*PutTrans*: 6, *PutDead*: 7), and within each population (*PutTrans*: 5 [Iowa], 2 [Mississippi]; *PutDead*: 3 [Iowa], 5 [Mississippi]), making these markers ideal for paternity assignment.

### *Solanum lycopersicum* markers

All NC4 plants were homozygous for resistant alleles at both the *Sw‐5* and *I‐3* genes, whereas the locally collected cherry tomato was homozygous for susceptible alleles at both markers, as expected (Appendices [Link], [Link], [Link]). The *Sw‐5*‐resistant allele (134 bp) was longer than the susceptible allele (108 bp) due to two indels (Table 4, Appendix S4), and the *I‐3*‐resistant allele (106 bp) was longer than the susceptible allele (94 bp) due to three indels (Table 4, Appendix S5). Furthermore, there were eight differentiating SNPs between resistant and susceptible *Sw‐5* alleles (Table 4, Appendix S4), and two differentiating SNPs between resistant and susceptible *I‐3* alleles (Table 4, Appendix S5). Although Li et al. (2018) and Samuel Hutton (personal communication) did not comment on SNPs between resistant and susceptible alleles, these differences are consistent with Dianese et al. (2010).

Further reducing the time and cost of paternity assignment, the size difference between resistant and susceptible alleles for both markers enabled us to differentiate between the two alleles by visualizing PCR products on 3% agarose gels (Appendix S6). Accordingly, we were able to differentiate between the allele in the experimental plants (resistant allele) and the allele in local populations (susceptible allele) without Sanger sequencing.

## RESULTS

### Pollen dispersal in experimental arrays

#### *Amaranthus tuberculatus*

**Figure 2:**
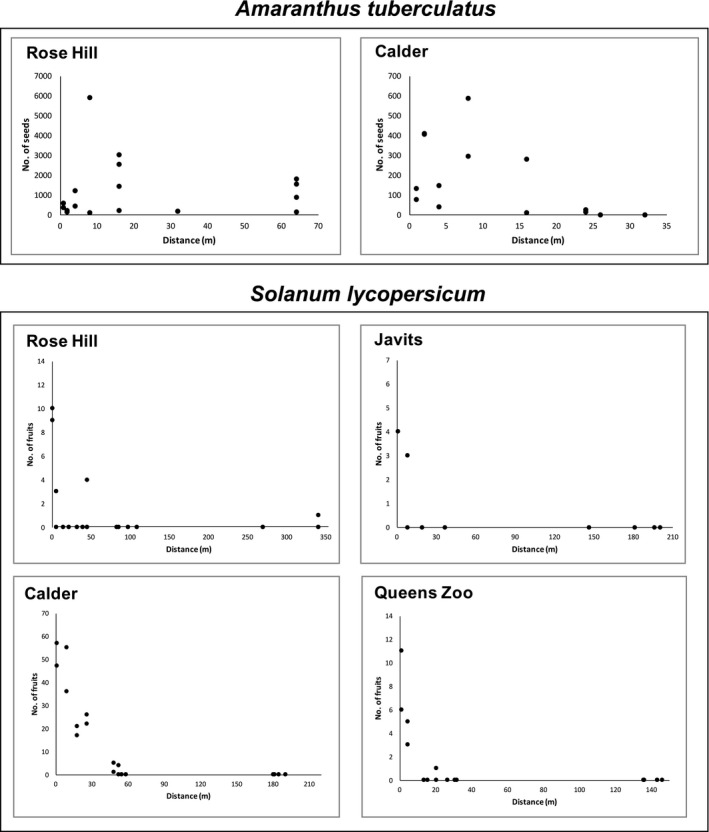
The number of *Amaranthus tuberculatus* seeds and number of *Solanum lycopersicum* fruits produced by pollen receptor plants at a given distance from the pollen donor group for each experimental array. Seed and fruit production were used to infer effective pollination by the pollen donor group. Paternity was assessed using two markers for each species on a representative subset of seeds or fruits. All tested *A*. *tuberculatus* seeds and 97% of *S*. *lycopersicum* fruits could be assigned to the grouped pollen donors using each of two markers; the two locally pollinated *S*. *lycopersicum* fruits are excluded from the figure.

A total of 23,187 *A. tuberculatus* seeds were produced between Rose Hill (20,776 seeds) and Calder (2411 seeds). The number of seeds produced by a given pollen receptor was used as a proxy for effective pollen dispersal and decreased with increasing distance from the pollen donor group at both sites (Fig. 2). To assess paternity in the two arrays, we genotyped 85 Rose Hill seeds and 95 Calder seeds at the ETS marker, and all contained the allele from the pollen donor group. We then genotyped 69 Rose Hill seeds and 25 Calder seeds at the *PutDead* marker and again found all contained the allele from the pollen donor group. Because all of the seeds we tested contained the pollen donor group allele for the two markers (ETS, *PutDead*), we did not genotype the seeds at the third marker (*PutTrans*). Furthermore, because all tested seeds contained the pollen donor group allele, and there were no known *A*. *tuberculatus* populations in the New York City metropolitan area, we inferred that all seeds produced in both arrays were the result of pollination by the pollen donor group. Pollen dispersal distance for each seed was then calculated as the Euclidean distance from the pollen donor group.

#### *Solanum lycopersicum*

**Figure 3:**
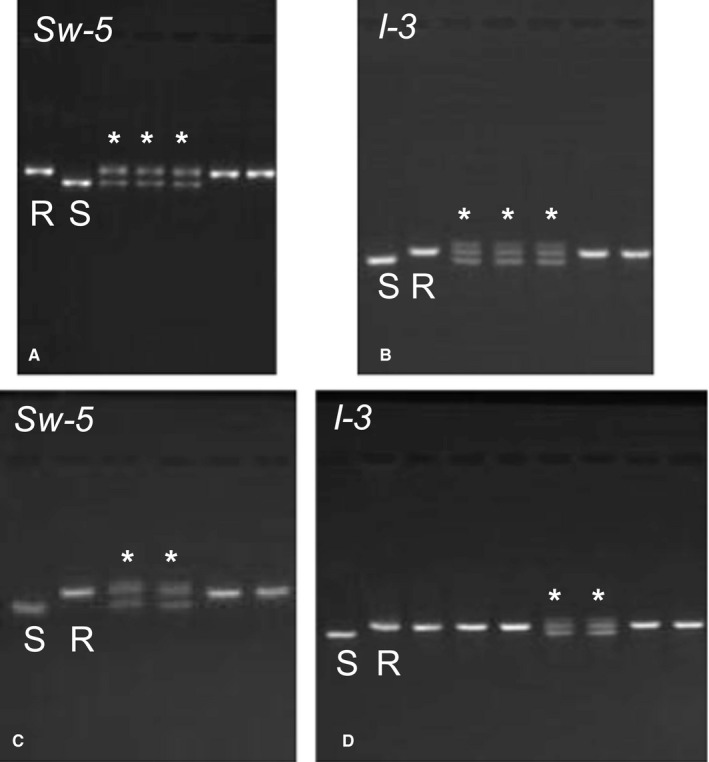
Results of the *Solanum lycopersicum* paternity assignment indicated a local pollen donor for three seeds from one fruit produced at the Rose Hill site (A, B) and two seeds from one fruit produced at the Queens Zoo site (C, D) at both genotyping markers (*Sw‐5* shown in A, C; *I‐3* shown in B, D). The first two lanes in each gel image are the homozygous‐resistant (R) and ‐susceptible (S) allele controls. Lanes with (*) show seeds that possessed both the R and S allele, suggesting a local pollen donor, which contributed the S allele.

A total of 355 *S. lycopersicum* fruits were produced across all four arrays (Rose Hill: 27 fruits, Calder: 291 fruits, Queens Zoo: 26 fruits, Javits: 11 fruits). The number of fruits produced by a given pollen receptor was used as a proxy for effective pollen dispersal and decreased with increasing distance from the pollen donor group at all four sites (Fig. 2). To assess paternity in the four arrays, we genotyped seeds from 21 Rose Hill fruits, 12 Calder fruits, 23 Queens Zoo fruits, and 11 Javits fruits at both the *Sw‐5* and *I‐3* markers. All seeds from Calder and Javits possessed only the resistant allele at both the *Sw‐5* and *I‐3* markers. Because all tested seeds at these two sites contained the pollen donor group allele, we inferred that all fruits produced in both arrays were the result of pollination by the pollen donor group. One fruit at Rose Hill and one fruit at Queens Zoo had seeds that possessed both the resistant and susceptible alleles at both markers, suggesting that they were pollinated by a local pollen donor (Fig. 3), we removed these from the pollen dispersal calculations (Fig. 2), as we were unable to determine the pollen source for these two fruits. We inferred that all untested fruits in both arrays were the result of pollination by the pollen donor group because we tested fruits from all plants in both arrays (accounting for 80–90% of fruits produced at these locations), detected local pollen in only one fruit at each array, and did not detect multiple paternity within any single fruit. Pollen dispersal distance for each fruit was then calculated as the Euclidean distance from the pollen donor group.

## DISCUSSION

We demonstrated here that, by using a combined method of physically grouped pollen donors and a single marker, we were readily able to assess pollen dispersal for both wind‐pollinated *A. tuberculatus* and insect‐pollinated *S. lycopersicum* after genotyping a limited number of pollen donors and a representative subset of offspring. This approach is faster and simpler than other approaches to assess pollen dispersal using paternity analysis, which typically entail genotyping all pollen donors, receptors, and offspring at several molecular markers. Our protocol was further simplified for *S. lycopersicum* because the size difference between the resistant alleles in the NC4 breeding line and the susceptible allele in local plants was detectable in the PCR product. Although we conducted this study using experimental arrays of pollen donor and receptor plants, this method could also be used in naturally occurring populations by identifying one or two pollen donors with rare alleles.

Through our protocol, all of the *A. tuberculatus* seeds we genotyped could be assigned paternity to the pollen donor group, indicating there were no local donors among the 180 seeds surveyed. A key reason that we did not detect local donors is presumably because we found no *A. tuberculatus* surrounding the experimental arrays. Similarly, the USDA Plants Database (USDA NRCS, 2017) and the New York Metropolitan Flora Project (Moore et al., 2002) contain no records of *A. tuberculatus* in the counties where our arrays were located. Additionally, we did not observe evidence of hybridization, although it has been shown to occur between *A. tuberculatus* and other *Amaranthus* species (e.g., *A. hybridus* L. [Trucco et al., 2005], *A. palmeri* S. Watson [Gaines et al., 2012]). Pollen dispersal in *Amaranthus* has only been observed at distances of up to 250 m in *A. palmeri* (Sosnoskie et al., 2012) and up to 800 m in *A. tuberculatus* (Liu et al., 2012). It is unclear whether dispersal at farther distances is possible in this species, as pollen dispersal in both studies was observed at the longest possible distance, given the study designs. Nonetheless, because our markers allowed differentiation between the two experimental populations (Iowa and Mississippi), which are separated by 1000 km, and our arrays were >1500 km from either of these populations, our protocol likely would have allowed us to differentiate local and experimental donors, had local donors been present.

The *S. lycopersicum* paternity assignment indicated that, out of the 66 fruits genotyped, only one fruit at Rose Hill and one fruit at the Queens Zoo showed both resistant and susceptible alleles, suggesting that seeds of these fruits were pollinated by local donors. This observation is not surprising because, during our survey of the landscape surrounding the *S. lycopersicum* arrays, we found *S. lycopersicum* growing concurrently at the New York Botanical Garden and Fordham University's community garden, both adjacent to our Rose Hill site. Similarly, several *S. lycopersicum* varieties were observed growing at residences near the Queens Zoo. The lack of local donors at the other two sites may also be related to location. Although Calder is located within suburban Westchester County, an area containing many gardens (Fetridge et al., 2008), the site is a private 0.5‐km^2^ research station and no other *S. lycopersicum* were growing at the station during the study period. Similarly, we did not locate *S. lycopersicum* in the area around the Javits array. At the time of the study, the Javits Center was relatively isolated from residences, gardens, and parks that could support *S. lycopersicum*, as it was bordered to the west by the Hudson River, to the south by the 0.11‐km^2^ West Side (Rail) Yard, and to the north by a Metropolitan Transit Authority bus depot.

### Applications in other systems

This combined approach can be applied to studying pollen dispersal in species with no locally occurring populations, as well as in naturally occurring populations in which rare alleles can be identified. As our results indicate, for species where no locally occurring populations are known, the use of rare alleles enables sampling a representative subset of seedlings to confirm no local pollen donors, and the grouping of pollen donors enables the use of the Euclidean distances between receptors and the donor group to estimate pollen dispersal patterns. This approach also worked for *S. lycopersicum*, which does grow in our area; the use of rare alleles, grouped pollen donors, and representative sampling of offspring enabled us to assess pollen dispersal patterns from the donor group after confirming no local pollen donors at two sites, and a single fruit with a local pollen source at two sites. Although this method requires optimization of species‐specific markers, the use of microsatellite markers also requires a similar type of optimization. Accordingly, our approach reduces the number of markers used to assign paternity, as well as the number of donors and offspring that need to be genotyped to assess pollen dispersal patterns.

This combined approach can also be utilized in natural populations. For example, studies on natural populations have identified one or two pollen donors with rare alleles and have mapped pollen dispersal from these donors (e.g., Müller, 1977; Stacy et al., 1996). Furthermore, pollen dispersal can be determined by measuring the distance from experimentally placed pollen donor(s) with the rare allele to plants that produce seeds with the rare allele, if plants in the population do not possess the rare allele. Additionally, although we used our experimental arrays to assess pollen dispersal within habitats, this method could be used to investigate pollen dispersal among habitats. Finally, this approach could be utilized to address other ecological questions, such as comparing male and female fitness in gynodioecious species or investigating multiple paternity.

## AUTHOR CONTRIBUTIONS

C.L.B. conceived of, designed, and executed this work under the advisement of B.Y.R., S.L.A., V.K.N., M.D.K.O., R.G.G., and J.D.L. All authors participated in writing and editing this article, gave final approval for this version to be submitted for publication, and agreed to be accountable for the work.

## Supporting information


**APPENDIX S1.** ETS region marker sequences from Iowa and Mississippi *Amaranthus tuberculatus* samples.

**APPENDIX S2.** Putative transmembrane 9 superfamily 4‐like protein (*PutTrans*) marker sequences from Iowa and Mississippi *Amaranthus tuberculatus* samples.

**APPENDIX S3.** Putative DEAD box ATP‐dependent RNA helicase (*PutDead*) marker sequences from Iowa and Mississippi *Amaranthus tuberculatus* samples.

**APPENDIX S4.** *Solanum lycopersicum–*resistant (R) and *–*susceptible (S) allele sequences for the *Sw‐5* marker using the *UF_Sw‐5* primer pair.

**APPENDIX S5.** *Solanum lycopersicum–*resistant (R) and *–*susceptible (S) allele sequences for the *I‐3* marker using the *I‐3_7g501* primer pair.

**APPENDIX S6.** *Solanum lycopersicum* PCR products for the *Sw‐5* marker using the *UF_Sw‐5* primer pair (A) and the *I‐3* marker using the *I‐3_7g501* primer pair (B) run on a 3% agarose gel at 150 V for 1.5 h and visualized under ultraviolet light.

## Data Availability

Marker sequences are archived in GenBank, and accession numbers for these sequences are provided in Table 4.
