# Supplementary material for: Combining rare alleles and grouped pollen donors to assign paternity in pollen dispersal studies
Source: Appl Plant Sci. 2020 Mar 4;8(3):e11330. doi: 10.1002/aps3.11330 (PMC7073328; doi:10.1002/aps3.11330)

DOI

APPENDIX S1. ETS region marker sequences from Iowa and Mississippi *Amaranthus tuberculatus* samples. Sequence analysis revealed only one genotype per population. Additionally, of the five SNPs (denoted by *) between the two populations, three were homozygous. The R and K in the sequences represent alternative nucleotides within that population (R = A/G, K = G/T).

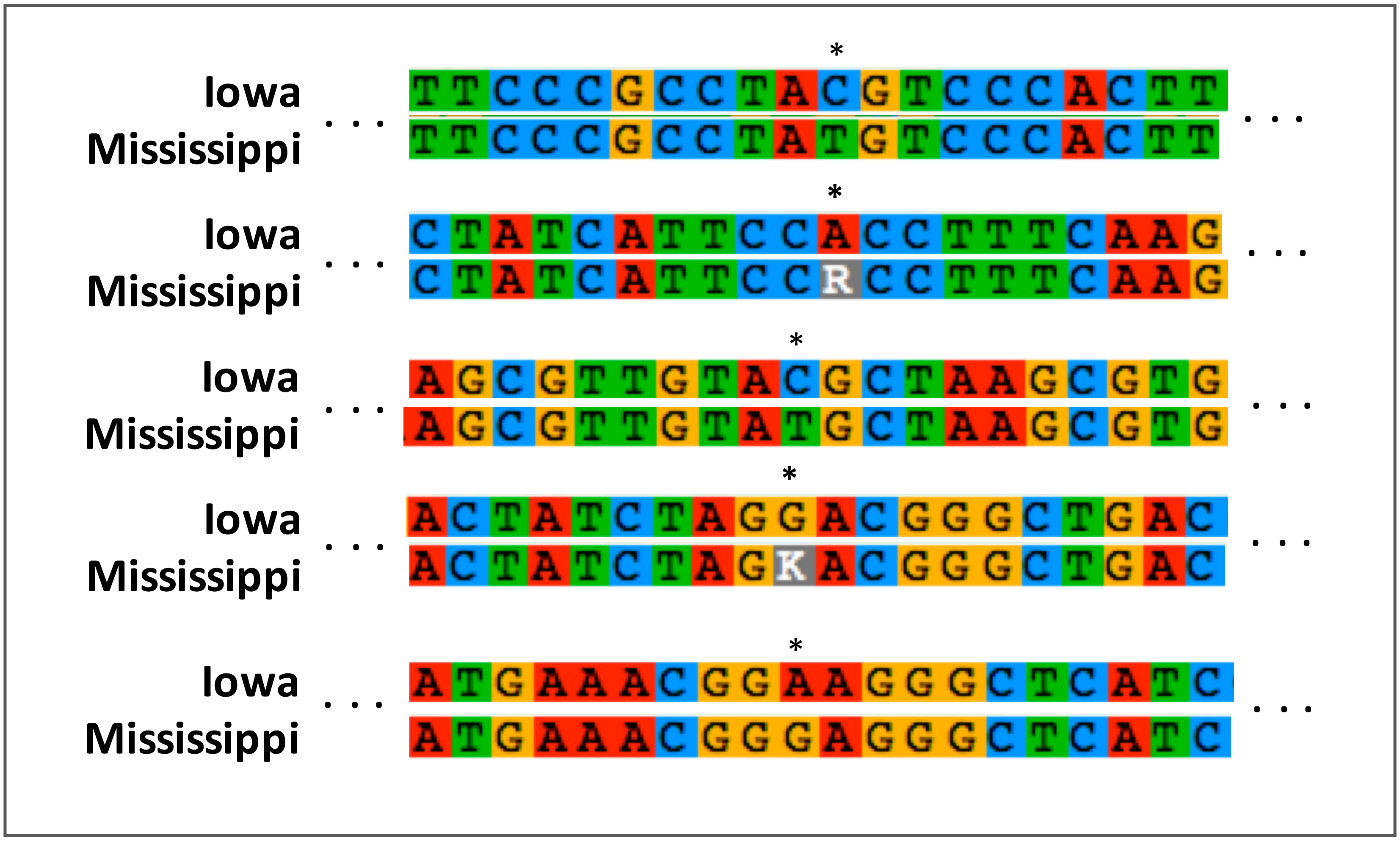

Supplement: Supplementary file 1 — APPENDIX S1. ETS region marker sequences from Iowa and Mississippi Amaranthus tuberculatus samples. [file APS3-8-e11330-s001.pdf]
