# Supplementary material for: Combining rare alleles and grouped pollen donors to assign paternity in pollen dispersal studies
Source: Appl Plant Sci. 2020 Mar 4;8(3):e11330. doi: 10.1002/aps3.11330 (PMC7073328; doi:10.1002/aps3.11330)

Butcher et al.—Applications in Plant Sciences 2020 8(3)—Data Supplement S2
DOI

APPENDIX S2. Putative transmembrane 9 superfamily 4-like protein (*PutTrans*) marker sequences from Iowa and Mississippi *Amaranthus tuberculatus* samples. Sequence analysis revealed three genotypes in the Iowa population (labeled #1–3) and four genotypes in the Mississippi population (labeled #1–4), but none of these genotypes were shared between the two populations. This marker also showed a manageable number of SNPs (denoted by *) between populations (6) and within each population (Iowa: 5, Mississippi: 2). The R, Y, and S in the sequences represent alternative nucleotides within that population (R = A/G, Y = C/T, S = C/G).

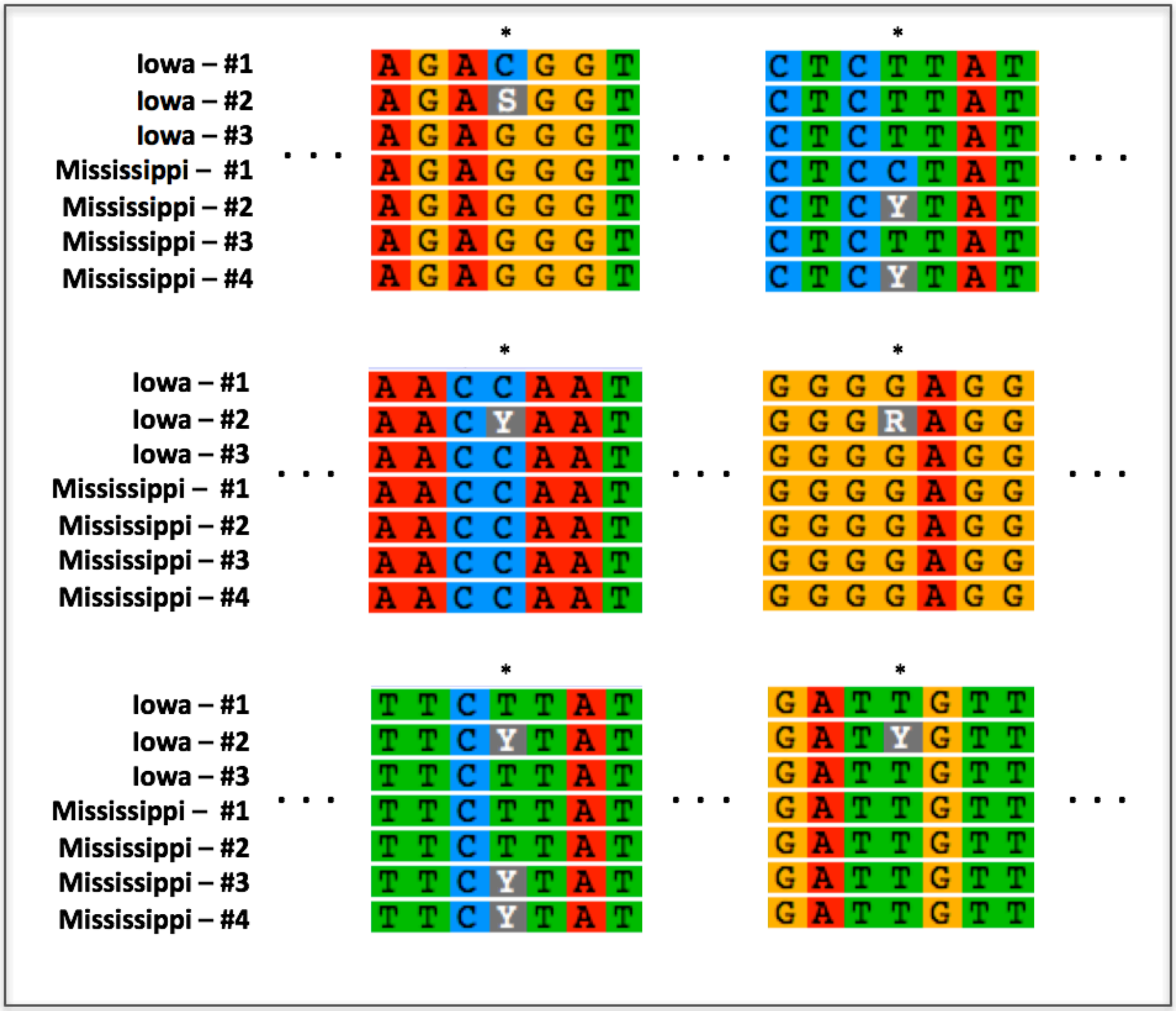

Supplement: Supplementary file 2 — APPENDIX S2. Putative transmembrane 9 superfamily 4‐like protein (PutTrans) marker sequences from Iowa and Mississippi Amaranthus tuberculatus samples. [file APS3-8-e11330-s002.pdf]
