# Supplementary material for: Combining rare alleles and grouped pollen donors to assign paternity in pollen dispersal studies
Source: Appl Plant Sci. 2020 Mar 4;8(3):e11330. doi: 10.1002/aps3.11330 (PMC7073328; doi:10.1002/aps3.11330)

Butcher et al.—Applications in Plant Sciences 2020 8(3)—Data Supplement S3
DOI

APPENDIX S3. Putative DEAD box ATP-dependent RNA helicase (*PutDead*) marker sequences from Iowa and Mississippi *Amaranthus tuberculatus* samples. Sequence analysis revealed four genotypes per population (labeled #1–4), and none of these genotypes were shared between the two populations. This marker also showed a manageable number of SNPs (denoted by *) between populations (7) and within each population (Iowa: 3, Mississippi: 5). The R, K, and W in the sequences represent alternative nucleotides within that population (R = A/G, K = G/T, W = A/T).

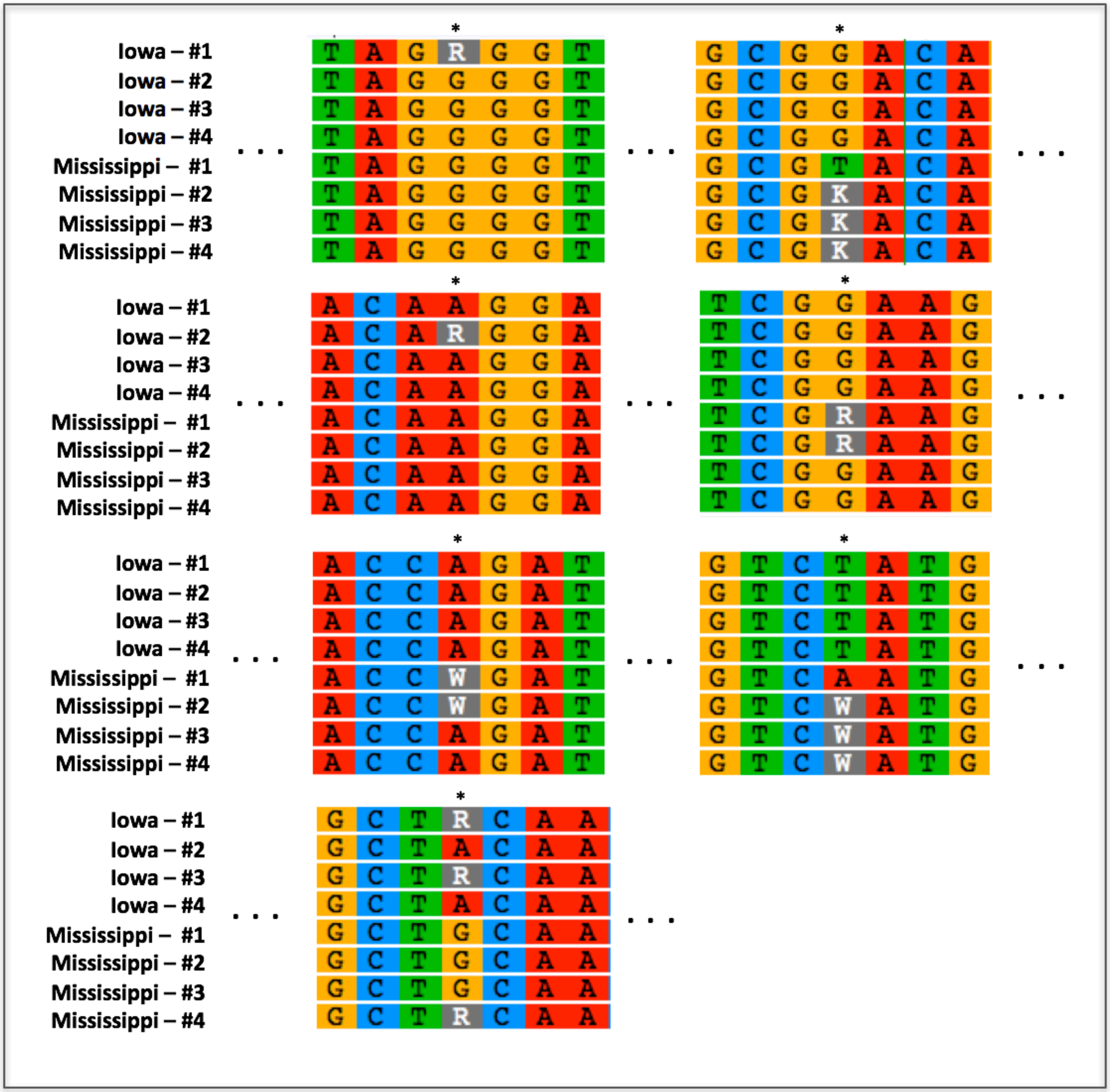

Supplement: Supplementary file 3 — APPENDIX S3. Putative DEAD box ATP‐dependent RNA helicase (PutDead) marker sequences from Iowa and Mississippi Amaranthus tuberculatus samples. [file APS3-8-e11330-s003.pdf]
