# Supplementary material for: Combining rare alleles and grouped pollen donors to assign paternity in pollen dispersal studies
Source: Appl Plant Sci. 2020 Mar 4;8(3):e11330. doi: 10.1002/aps3.11330 (PMC7073328; doi:10.1002/aps3.11330)

APPENDIX S4. *Solanum lycopersicum*–resistant (R) and –susceptible (S) allele sequences for the *Sw-5* marker using the *UF_Sw-5* primer pair. The R allele is 26 bp longer than the S allele due to two indels (labeled 1 and 2), and there were also eight SNPs (denoted by *) between the two alleles. All NC4 *S. lycopersicum* leaf tissue samples were homozygous for the R allele, and the leaf tissue sample taken from the cherry *S. lycopersicum* cultivar from the New York Botanical Garden was homozygous for the S allele.

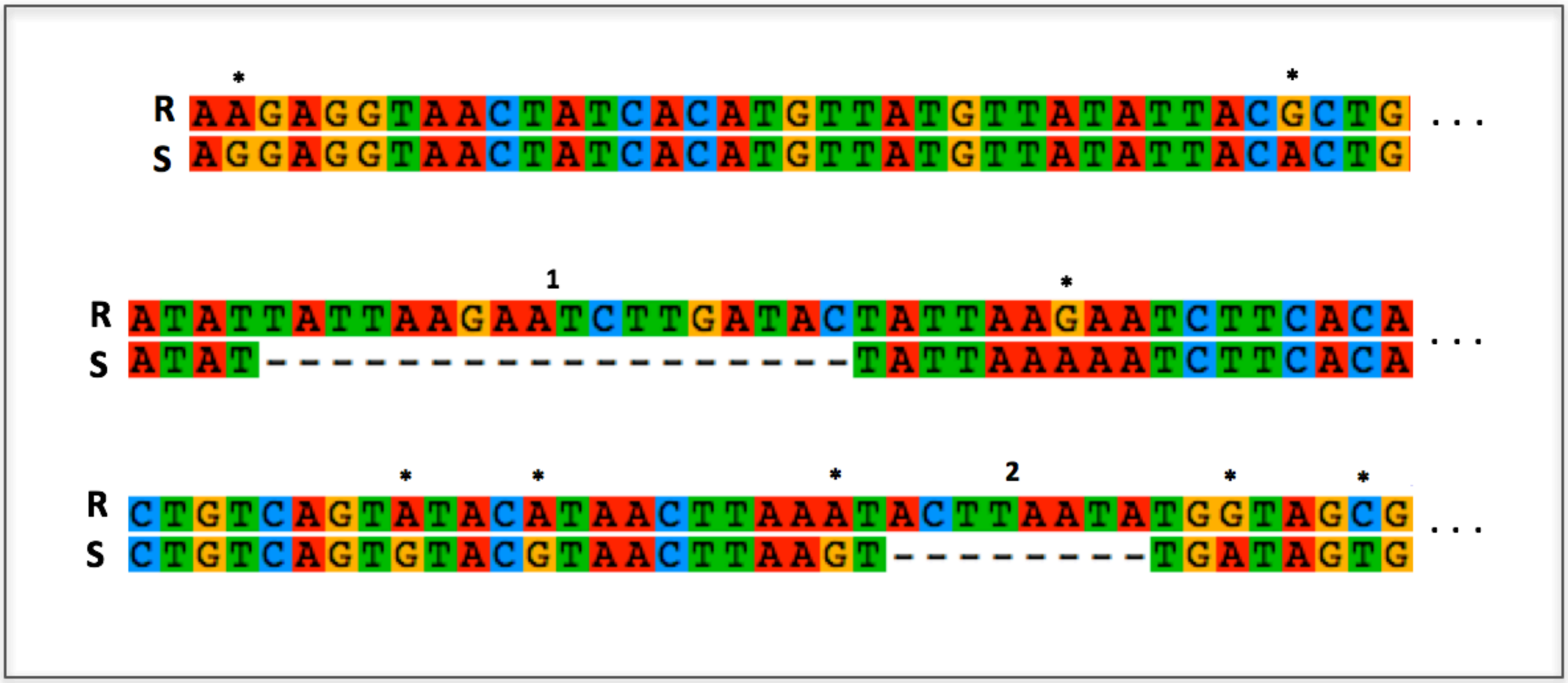

Supplement: Supplementary file 4 — APPENDIX S4. Solanum lycopersicum–resistant (R) and –susceptible (S) allele sequences for the Sw‐5 marker using the UF_Sw‐5 primer pair. [file APS3-8-e11330-s004.pdf]
