# Supplementary material for: Combining rare alleles and grouped pollen donors to assign paternity in pollen dispersal studies
Source: Appl Plant Sci. 2020 Mar 4;8(3):e11330. doi: 10.1002/aps3.11330 (PMC7073328; doi:10.1002/aps3.11330)

Butcher et al.—Applications in Plant Sciences 2020 8(3)—Data Supplement S5
DOI

APPENDIX S5. *Solanum lycopersicum*–resistant (R) and –susceptible (S) allele sequences for the *I-3* marker using the *I-3_7g501* primer pair. The R allele is 12 bp longer than the S allele due to three indels (labeled 1–3), and there were also two SNPs (denoted by *) between the two alleles. All NC4 *S. lycopersicum* leaf tissue samples were homozygous for the R allele, and the leaf tissue sample taken from the cherry *S. lycopersicum* cultivar from the New York Botanical Garden was homozygous for the S allele.

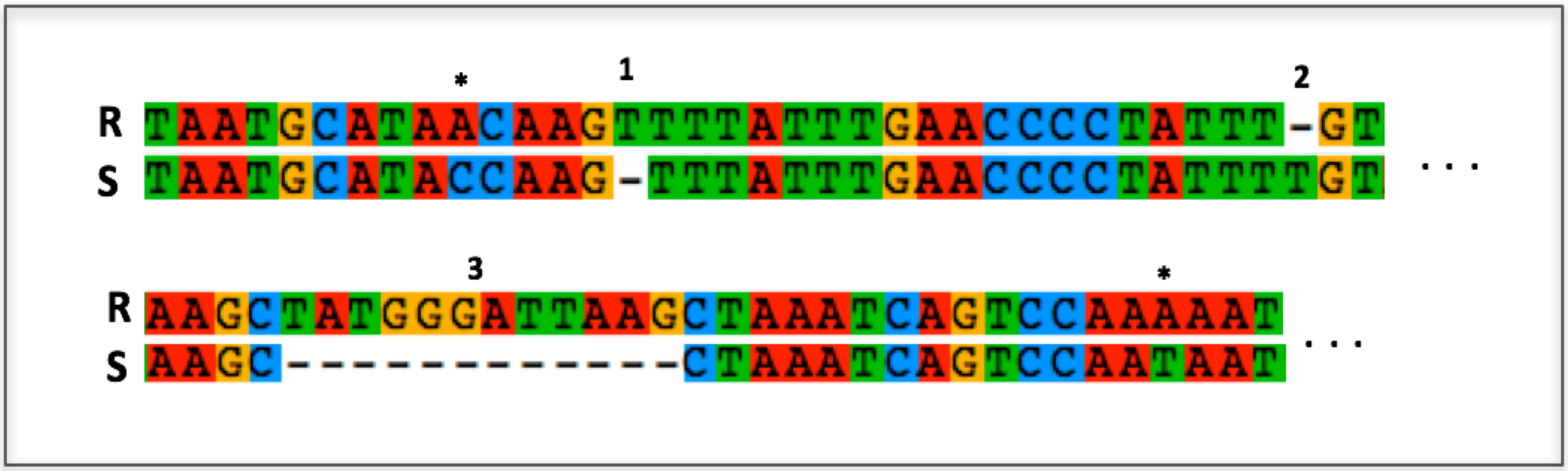

Supplement: Supplementary file 5 — APPENDIX S5. Solanum lycopersicum–resistant (R) and –susceptible (S) allele sequences for the I‐3 marker using the I‐3_7g501 primer pair. [file APS3-8-e11330-s005.pdf]
