# Supplementary material for: Combining rare alleles and grouped pollen donors to assign paternity in pollen dispersal studies
Source: Appl Plant Sci. 2020 Mar 4;8(3):e11330. doi: 10.1002/aps3.11330 (PMC7073328; doi:10.1002/aps3.11330)

APPENDIX S6. *Solanum lycopersicum* PCR products for the *Sw-5* marker using the *UF_Sw-5* primer pair (A) and the *I-3* marker using the *I-3_7g501* primer pair (B) run on a 3% agarose gel at 150 V for 1.5 h and visualized under ultraviolet light. For both images, the first and last wells were loaded with 5 µL of a 100-bp ladder (Promega Corporation). Negative controls were loaded in wells labeled NC. The NC4 *S. lycopersicum* PCR products were loaded in wells 1–9 and 11–19 (homozygous for the resistant allele), and the New York Botanical Garden cherry *S. lycopersicum* PCR product was loaded in well 10 (homozygous for the susceptible allele). The *Sw-5*-resistant allele is 26 bp longer than the susceptible allele, due to two indels (A), and the *I-3*-resistant allele is 12 bp larger than the susceptible allele, due to three indels (B).

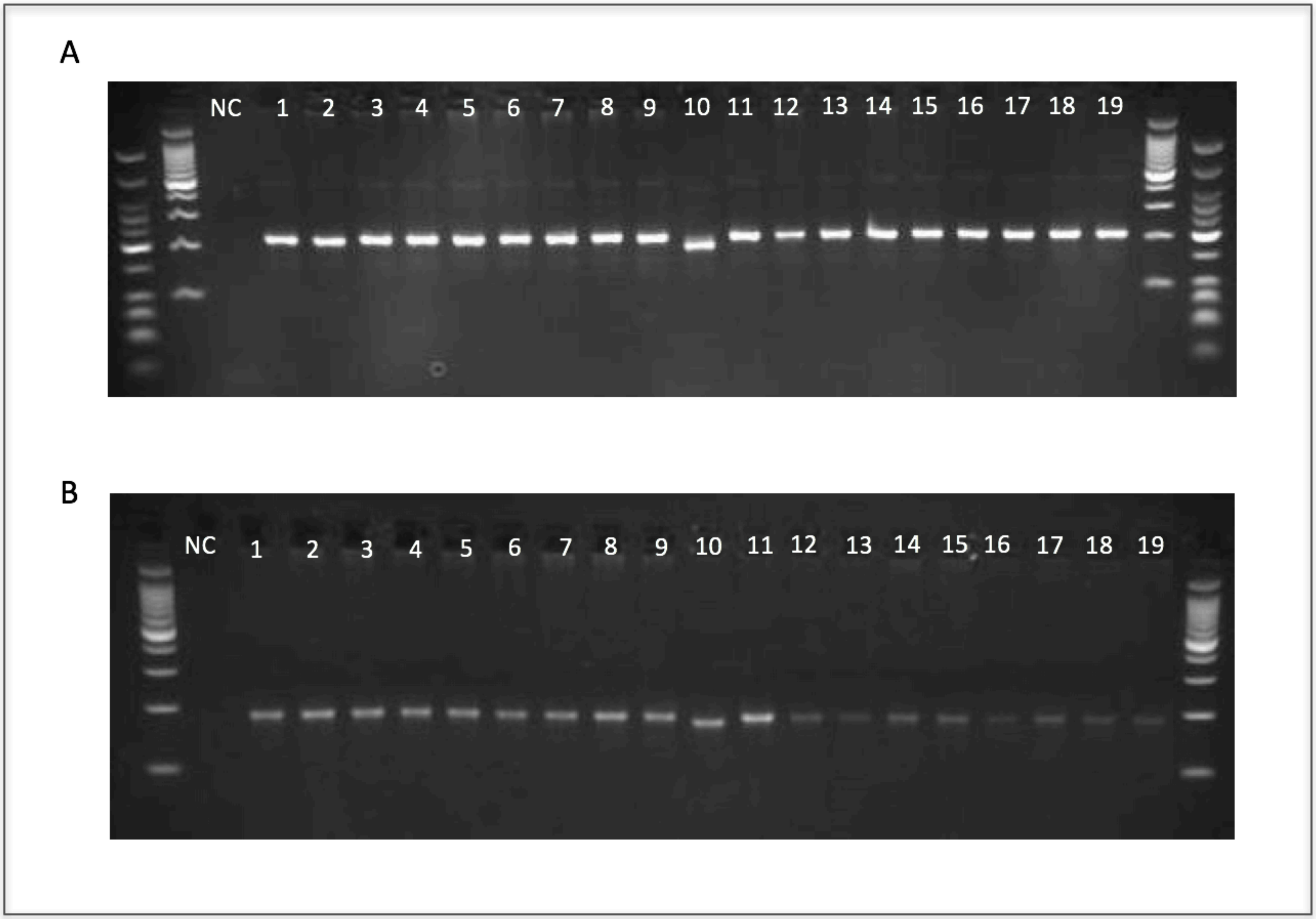

Supplement: Supplementary file 6 — APPENDIX S6. Solanum lycopersicum PCR products for the Sw‐5 marker using the UF_Sw‐5 primer pair (A) and the I‐3 marker using the I‐3_7g501 primer pair (B) run on a 3% agarose gel at 150 V for 1.5 h and visualized under ultraviolet light. [file APS3-8-e11330-s006.pdf]
